# Developing a surveillance system for HIV pre-exposure prophylaxis (PrEP) use in pregnancy in Malawi

**DOI:** 10.1186/s12884-026-09190-2

**Published:** 2026-05-08

**Authors:** Funny Kamanga, Madeleine A. Squibb, Watipa Nyangulu, William Wu, Jiehua Chen, Chatonda Ngwira, Anna Drabko, Wiktor Garbarek, Maciej Pomykala, Dominik Bilicki, Charity Nakanga, Gladwell Masiye, Lameck Chinula, Tisungane Mvalo, Maganizo B. Chagomerana, Mitch M. Matoga, Wiza Kumwenda, Tawonga Mkochi, Ernest Matola, Peter Dias, Nomthata Mkochi, Virginia Thonyiwa, Rose K. Nyirenda, Linley Chewere, Washington Ozituosauka, Andreas Jahn, Joe Nkhonjera, James Odek, Deborah Hoege, Sheree Schwartz, Felluna Chauwa, Sufia Dadabhai, Sam Phiri, Sarah E. Rutstein, Michael Herce, Irving Hoffman, Sara Allinder, Charles B. Holmes, Mina C. Hosseinipour, Friday Saidi

**Affiliations:** 1 University of North Carolina (UNC) Project Malawi, Lilongwe, Malawi; 2 Department of Epidemiology, Gillings School of Global Public Health, Chapel Hill, North Carolina USA; 3 Quantitative Engineering Design (QED), Lilongwe, Malawi; 4 Department of State-President’s Emergency Plan for AIDS Relief, United States Government, Lilongwe, Malawi; 5 World Vision Malawi, Lilongwe, Malawi; 6 Department of HIV, STI and Viral Hepatitis, Ministry of Health Malawi, Lilongwe, Malawi; 7 Center for Innovation in Global Health, Georgetown University, Washington, DC USA; 8 Department of Epidemiology, Johns Hopkins Bloomberg School of Public Health, Baltimore, MD USA; 9 Johns Hopkins Research Project (JHP), Kamuzu University of Health Sciences, Blantyre, Malawi; 10 Partners in Hope (PIH), Lilongwe, Malawi; 11 Institute for Global Health and Infectious Diseases, University of North Carolina at Chapel Hill, Chapel Hill, NC USA

**Keywords:** HIV Prevention, Pre-Exposure Prophylaxis, Pregnancy Exposure Registry, Pharmacovigilance in pregnancy, Long-Acting Injectable PrEP

## Abstract

**Background:**

Pregnant and breastfeeding women are at elevated risk of HIV acquisition. The World Health Organization (WHO) recommends use of oral TDF/FTC and long-acting injectables cabotegravir (CAB-LA) and lenacapavir (LEN) for HIV pre-exposure prophylaxis (PrEP) in pregnancy. However, safety data for emerging regimens remain limited due to the exclusion of pregnant women from early clinical trials and insufficient routine, program-embedded surveillance, especially in resource-constrained settings. In March 2024, as Malawi introduced CAB-LA, the PrEP Pregnancy Registry was established to generate real-world safety data on PrEP exposure during pregnancy.

**Methods:**

The PrEP Pregnancy Registry provides prospective safety surveillance of pregnant women on PrEP within routine service delivery across Malawi. Guided by the WHO collaborative framework for antiretroviral safety in pregnancy, the Registry was established through a phased process involving the Malawi Ministry of Health, research and implementing partners. Initially implemented at five CAB-LA early-access facilities, it was refined with provider feedback and expanded to 30 additional facilities. The registry utilizes ScanForm, an AI-powered tool that uses optical character recognition technology to digitize and analyze handwritten data. Birth outcomes are measured using WHO’s harmonized pharmacovigilance indicators for maternal and infant birth outcomes.

**Discussion:**

The Registry provides a scalable model for real-world pharmacovigilance of PrEP use in pregnancy. The use of ScanForm technology maximizes data quality and ensures integration with existing systems. Resulting evidence will fill critical gaps in PrEP safety data during pregnancy and inform national and global policies for integrating CAB-LA and emerging PrEP regimens into routine maternal health care settings.

**Trial Registration Numbers:**

The Pregnancy, Infant and Maternal Health Outcomes (PrIMO) sub-study within the Preventing Infant Infection with Implementation Science in Malawi (PRI^3^SM) was registered in ClinicalTrials.gov on 05th December 2023 with trial registration number NCT06158126, and the Path to Scale study was registered on 29th March 2024 with trial registration number NCT06319105.

## Background

An estimated 120,000 children acquired HIV globally in 2024, with most infections attributable to vertical transmission in Southern Africa [1]. Pregnant and lactating women in this region, including Malawi, bear a disproportionately high burden of HIV. The risk of acquisition nearly doubles during pregnancy and the postpartum period due to a combination of biological factors—such as hormonal and immunological changes that increase susceptibility—and socio-economic and behavioral factors that impact vulnerability [2–5].

The World Health Organization (WHO) currently recommends oral tenofovir disoproxil fumarate/emtricitabine (TDF/FTC) in fixed-dose combination, the dapivirine vaginal ring (DVR), intramuscular long-acting injectable cabotegravir (CAB-LA), and, more recently, subcutaneous long-acting lenacapavir (LEN) as options for HIV pre-exposure prophylaxis (PrEP) [6, 7] during pregnancy and breastfeeding [7, 8]. While the safety of oral TDF/FTC is well-established, safety data for emerging modalities, such as CAB-LA, remain limited [6, 9]. Most early-stage studies exclude pregnant populations, contributing to delays in access due to ongoing safety uncertainty [5, 9–14]. Expanding access to injectable options among pregnant and breastfeeding women is critical, as the less frequent dosing schedule compared to oral PrEP helps mitigate adherence and stigma challenges [15].

Maternal pharmacovigilance—systematic safety monitoring of medications during pregnancy and lactation—through large-scale surveillance programs is essential to identify rare adverse outcomes [10, 15, 16]. Longitudinal surveillance is particularly important for long‑acting injectables, such as CAB, which have a longer half‑life – approximately 6 to 12 weeks following intramuscular injection than oral PrEP which has a half-life approximately 41 h after oral dosing [17, 18]. Tracking the timing of drug exposure during pregnancy is another critical component of effective risk assessment [8, 16, 19–23]. Improved surveillance is especially necessary in low- and middle-income countries (LMICs) like Malawi, where adverse pregnancy outcomes including preterm birth are more prevalent and pregnancy exposure registries remain scarce [24].

Malawi is at a pivotal moment in its HIV prevention strategy, having introduced injectable CAB-LA in March 2024. As part of this initiative, the PrEP Pregnancy Registry was developed by Quantitative Engineering Design (QED) in collaboration with research partners and the Ministry of Health to generate evidence on the safety of PrEP use in pregnancy. The Registry is currently being piloted and evaluated as part of the Pregnancy, Infant and Maternal Health Outcomes (PrIMO, NCT06158126) sub-study within the Preventing Infant Infection with Implementation Science in Malawi (PRI^3^SM) and Path to Scale (NCT06319105) studies prior to nationwide expansion. It prospectively captures PrEP exposure and birth outcomes, enabling continuous safety monitoring, including the detection of rare adverse events, among pregnant women receiving oral TDF/FTC or TDF/3TC, injectable CAB-LA, and, as access expands, long-acting lenacapavir (LEN) and other agents. By embedding data collection within routine maternal care services, the registry provides continuous, real-world surveillance for established and emerging PrEP modalities. This paper describes the process of developing and piloting the PrEP Pregnancy Registry in Malawi.

## Methods

### Conceptual framework

**Fig. 1:**
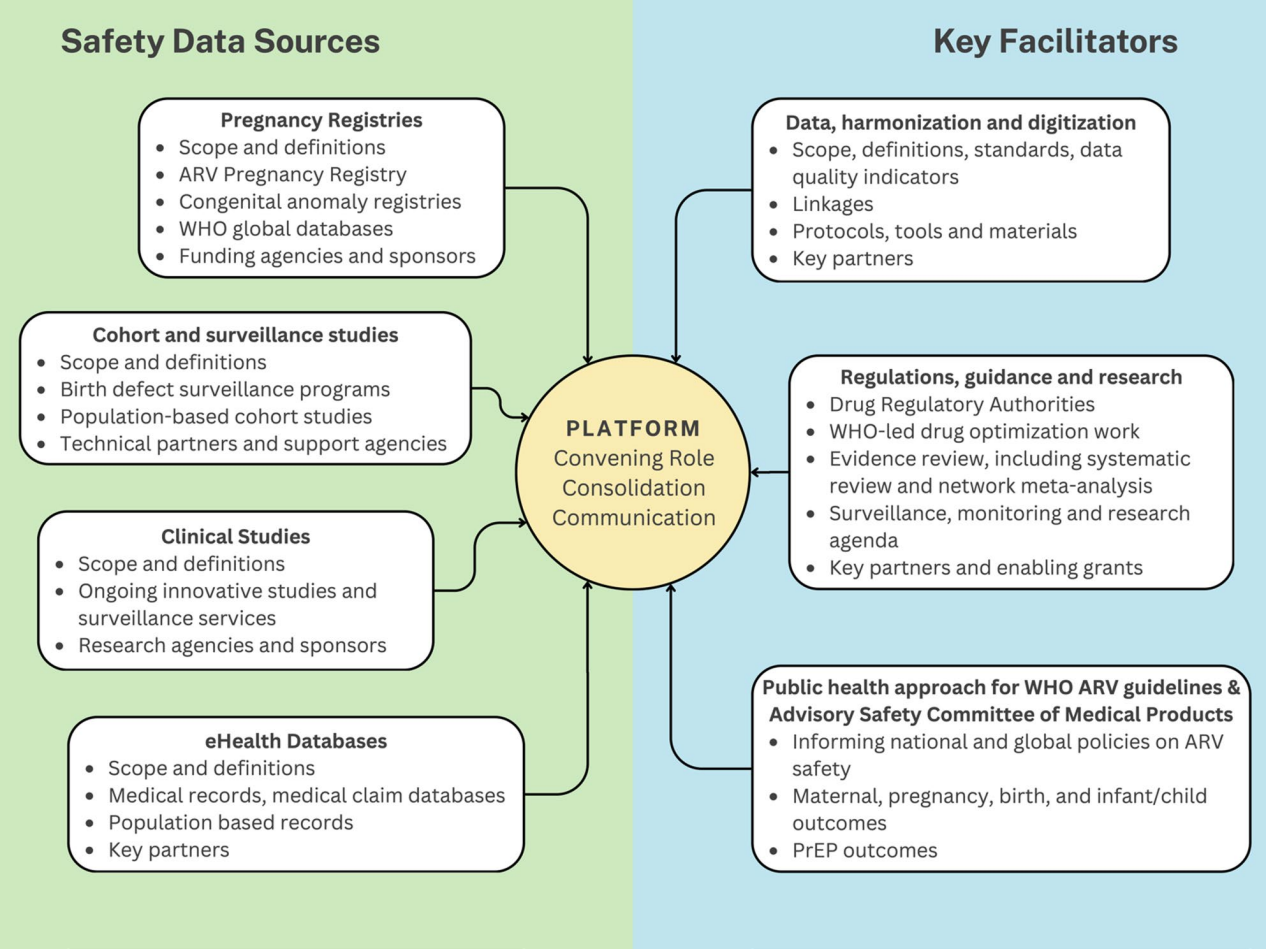
Replicated version of WHO’s collaborative conceptual framework for active surveillance of ARV safety in pregnancy [25].

The design of the PrEP Pregnancy Registry was informed by the WHO collaborative conceptual framework for surveillance of the safety of antiretroviral drugs in pregnancy [25]. The framework, illustrated in Fig. 1, is guided by core principles of standardization, data quality, and harmonization of outcomes. These principles facilitate collaboration and interoperability across surveillance networks and support the rapid evaluation of any identified safety signals [25, 26]. In accordance with these principles, the Registry was collaboratively designed with key HIV program partners and utilizes innovative technology to ensure data quality and harmonization with existing systems.

### Registry development process

The Registry was developed through a phased, collaborative approach including: (i) needs assessment, (ii) stakeholder identification and engagement, (iii) feasibility assessment, (iv) Registry design, (v) pilot implementation and iterative refinement and (iv) implementation evaluation.

### Needs assessment

Prior to the Registry, Malawi’s pharmacovigilance mechanisms for PrEP focused on uptake and adherence, with no systematic monitoring of pregnancy and infant outcomes. Robust evidence of the safety of PrEP during pregnancy is still being established globally [27, 28]. This assessment emphasized the need for a dedicated registry to monitor maternal and infant outcomes, particularly following the approval and recommended use of injectable CAB-LA in pregnancy [8, 16, 19, 25, 26, 29].

### Stakeholder identification and engagement

The Registry was developed in partnership with the Malawi Ministry of Health and Sanitation (MoH) and key stakeholders to ensure alignment with national HIV priorities and harmonization with existing PrEP programs and tools. Design was led by the University of North Carolina (UNC) Project Malawi under the NIH-funded PRI^3^SM grant and the Gates Foundation-funded Path to Scale study consortium, with consultation from QED, the registry developer. Key stakeholders within the Path to Scale consortium include the MoH (Department of HIV/AIDS, STIs, and Viral Hepatitis) and major HIV PEPFAR implementing partners. Research partners include UNC, Johns Hopkins University, and Georgetown University’s Center for Innovation in Global Health (CIGH). All partners were engaged based on HIV program expertise. A series of consultative meetings were conducted to refine registry objectives and outcome measures.

### Feasibility assessment

A feasibility assessment evaluated the technical, operational, financial and political viability of implementing the registry. The MoH selected QED to develop the registry using ScanForm technology, which has been effectively used within Malawi’s PrEP and HIV Testing Services (HTS) and across several African countries including Kenya, Namibia, Tanzania and Nigeria [30]. This decision ensured integration with current systems, familiarity with the technology, and transfer of skills when facility staff are re-assigned across HIV services. Operational feasibility included assessing staffing and training needs at CAB-LA PrEP delivery sites. The registry was also aligned with updated PrEP guidelines from the Ministry of Health [31].

### Registry design

The PrEP Pregnancy Registry was designed as a prospective, programmatic exposure registry that passively enrolls pregnant PrEP users during routine maternal health care. The passive enrollment approach ensures integration within the continuum of care, allowing for the evaluation of real-world implementation and scalability. This is particularly relevant, as oral TDF/FTC or TDF/3TC and injectable CAB-LA are already approved for use in Malawi. However, given the limited evidence on the safety of CAB-LA use during pregnancy, early national guidelines did not permit CAB-LA initiation during pregnancy outside of a dedicated safety cohort. Therefore, the Registry employs a hybrid design involving a prospective cohort at Bwaila District Hospital in Lilongwe established under the PRI^3^SM-PrIMO study. Women in this cohort are enrolled during pregnancy and followed up, with their infants, through one year postpartum. Written informed consent is obtained for participation in the safety cohort, and the target sample size is 621 mother-infant pairs [29]. For the broader Registry, individual consent is not required, though women are asked to voluntarily provide contact information to facilitate follow-up. For both components, inclusion in the registry is contingent upon confirmed pregnancy and meeting PrEP eligibility criteria per the Malawi national PrEP guidelines [31]. This design ensures enrollment prior to the pregnancy outcome, minimizing bias in outcome assessment.

In alignment with the WHO framework on data harmonization (Fig. 1), and consistent with the WHO-recommended standardized pharmacovigilance indicators for monitoring maternal, birth, and infant outcomes, the Registry includes the following key pregnancy outcome measures: (i) live birth at term, defined as delivery of a live-born infant at ≥ 37 completed weeks of gestation; (ii) preterm live birth, defined as delivery of a live-born infant before 37 completed weeks of gestation; (iii) fresh stillbirth, defined as the birth of an infant with no signs of life after 28 weeks of gestation, without features of maceration, indicating death during labor or delivery; (iv) macerated stillbirth, defined as the birth of an infant with no signs of life after 28 weeks of gestation, accompanied by degenerative changes consistent with intrauterine death before the onset of labor; and (v) spontaneous miscarriage, defined as expulsion of a non-viable pregnancy before 28 completed weeks of gestation, occurring in the absence of medical or surgical termination [25].

PrEP drug information – exposure to oral TDF/FTC or TDF/3TC or injectable CAB-LA is acquired through linkage to national PrEP service delivery data with a unique PrEP ID assigned to each participant. This design provides flexibility in the exposure variable, allowing the Registry to easily adapt to new PrEP modalities, including LEN, without requiring changes to the paper forms. PrEP data sources also utilize ScanForm technology, undergo similar data quality checks, and are available through the same ScanForm portal. This harmonization facilitates record linkage across data sources. The number of unlinked records is tracked and displayed on the QED data dashboard, and study staff follow up with sites directly. Additionally, an HTS link ID is issued at each PrEP visit, which links clients to the HTS register, as HIV testing is a prerequisite for PrEP initiation or continuation, including refills and follow-up injections.

### Data collection procedures and management

**Fig. 2:**
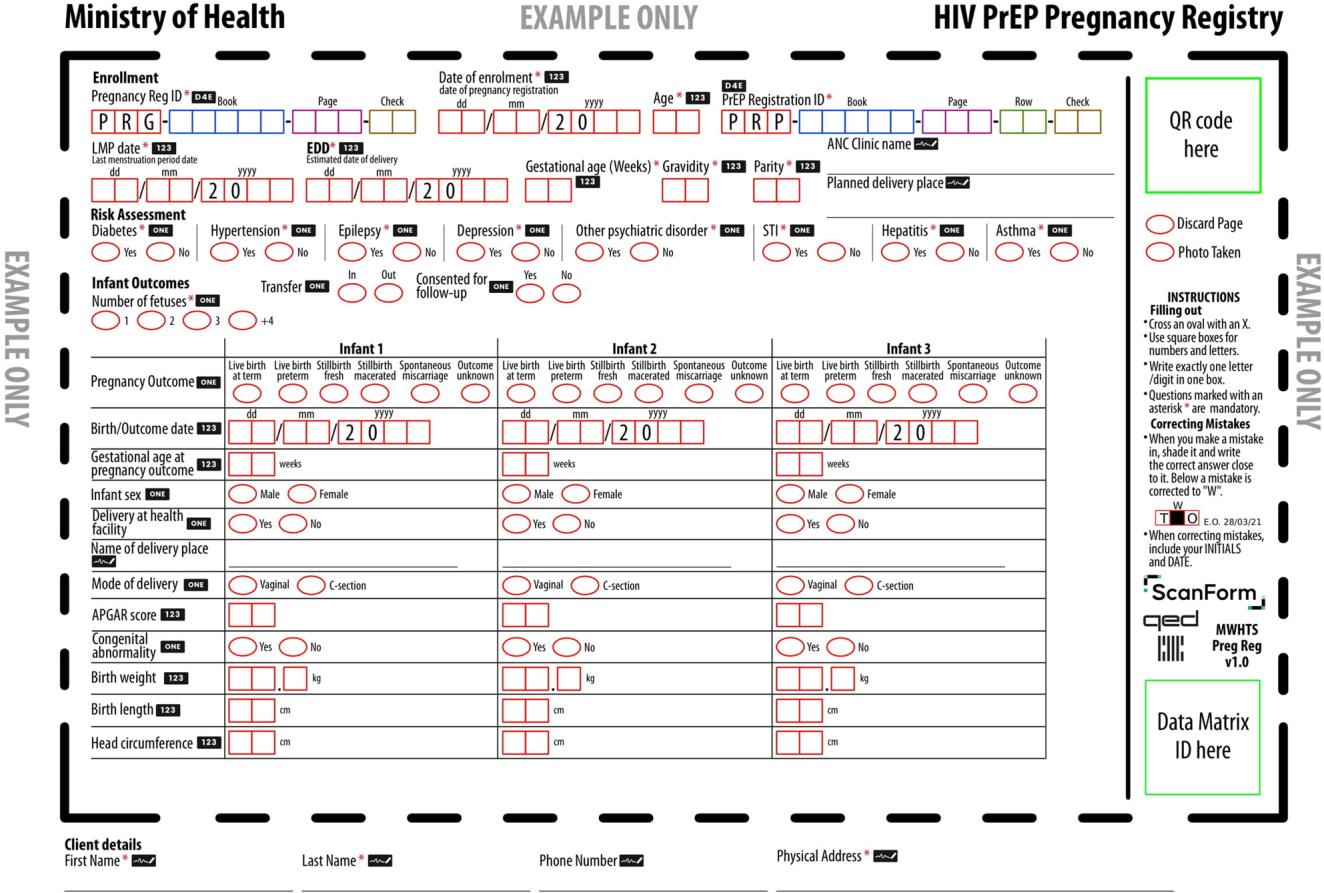
Example of the ScanForm PrEP Pregnancy Registry

Data are initially collected on a paper-based tool, shown in Fig. 2 below. Structured sections include participant identification at enrollment, pregnancy details, relevant covariates such as comorbid conditions, and maternal and infant outcomes. Information for these sections will primarily draw from medical records and routinely collected data from ANC and PrEP delivery sites.

These data are subsequently uploaded to the MoH database with ScanForm technology, which utilizes the optical character recognition (OCR) to digitize and analyze handwritten data efficiently and securely [30].

The digitization process follows four steps: (i) completion of the paper-based register, (ii) photo capture via Android device, (iii) AI-powered digitization upon photo upload, and (iv) real-time access to data and visualizations through the ScanForm portal. A key feature of this technology is the automated feedback function that produces near immediate data queries for missing data or logic patterns. Any data quality issues are sent to the original submission phone and are available for download from the ScanForm portal. This portal displays Registry data in real-time, allowing for regular monitoring of data quality. Providers correct issues on the original paper forms and retake the photo, and the record is reuploaded with the most recent information. At submission, all sections of the Registry are required except pregnancy outcomes, to allow for data quality assurance at enrollment when outcomes are not yet available.

Data collection is conducted by trained registry administrators such as PrEP providers (clinicians, nurses, or midwives) at two time points: (1) registry enrollment following pregnancy confirmation and PrEP prescription, and (2) outcome information at the time of delivery or remotely, if delivery does not happen in the facility. This approach accommodates women who access PrEP, attend antenatal care (ANC) or deliver at different facilities, maintaining continuity across the care spectrum and consistent outcome documentation. To ensure that all birth outcomes are recorded estimated delivery date (EDD) is used to prompt follow-up of women with missing outcomes past their estimated date. At each timepoint, an updated OCR image will be captured, updating prior records to retain the most current data.

All personally identifiable information (PII) will be stored securely on locally hosted servers in Malawi. Access to PII will be restricted to clinical providers, data clerks, and designated evaluation staff, with strict access controls in place. Only aggregate, de-identified data will be available to the research team, as managed by QED. All data handling will adhere to Malawi’s data protection laws, MoH policies, and established standard operating procedures (SOPs).

### Pilot implementation and iterative refinement

The Registry rollout was designed as a phased implementation to allow for interim evaluation and refinement. Before implementation, the Malawi National PrEP Guidelines were revised to incorporate CAB-LA and related data collection tools, including the PrEP Pregnancy Registry [31]. Updates were led by MoH to ensure alignment with national PrEP programming. A Training of Trainers (ToT) was conducted for the two districts in the pilot phase. The ToT led by MoH Department of HIV/AIDS and QED, the technical lead for the OCR system. The ToT provided an opportunity for capacity building and structured feedback on the registry tools, which informed revisions to improve usability and integration into clinical workflows.

Following the ToT, trainings were conducted for PrEP providers at six CAB-LA early-access implementation sites. Participants were trained to integrate PrEP documentation tools into routine service delivery. Paper OCR registers and Android mobile phones – programmed to run the ScanForm application only – were provided by QED and designated as MoH property. Five of those sites served as early phased implementation sites for the PrEP Pregnancy Registry, supporting iterative refinement of tools and processes. The sixth site was excluded, as their target clientele is men who have sex with men.

The Registry was implemented at those five sites in March 2024 and subsequently expanded to 30 more sites, to include 35 of the 36 sites that served as early access points for injectable CAB-LA PrEP under the Path to Scale implementation science study. These 35 sites include 20 facilities in Blantyre and 15 in Lilongwe, the two most populous cities in Malawi. Selection was guided by MoH guidelines, which prioritized high burden, urban districts. All selected facilities already offered oral PrEP and had high client volume, services for key populations, and existing implementing partner presence. Implementation at these sites constitutes the ‘pilot phase’, which will continue through March 2026, followed by national rollout.

### Registry evaluation

Registry data, including enrollments and birth outcomes, are available on a dashboard located on the Malawi HTS portal as soon as they are uploaded by QED. MoH, QED and Path to Scale staff provide supportive supervision to the sites to ensure timely resolution of data quality issues, including follow-up with women missing pregnancy outcomes. Formal analysis of all birth outcomes will be conducted as part of the PRI^3^SM-PrIMO study.

The pilot phase of the Registry implementation will be evaluated at 6-, 12-, 18- and 24-months post-initial-initiation (March 2024) to guide nationwide expansion to all PrEP delivery sites. Implementation will be assessed using three key indicators: (i) adoption, defined as the number and proportion of sites that have initiated Registry use; (ii) reach, defined as the total number of entries and site-specific entries, disaggregated by PrEP modality; and (iii) fidelity, defined as the number and proportion of entries with complete information on required infant and obstetric outcomes. Required outcomes include the number of fetuses and, for each fetus, documentation of pregnancy outcome and presence or absence of congenital anomalies. Facilities will receive structured feedback on data quality, including levels of missingness, unmatched PrEP IDs, and mismatched or incomplete PrEP modality records, to support continuous improvement.

## Discussion

The PrEP Pregnancy Registry addresses gaps in surveillance of PrEP usage in pregnancy, especially for long-acting injectable PrEP, in Malawi and globally [6, 9]. It builds upon previous pregnancy exposure registries, including the Antiretroviral Pregnancy Registry (APR), which has provided critical data on antiretroviral exposures through international, voluntary reporting systems, and pilot registries in Botswana, South Africa and Zambia have demonstrated the feasibility of embedding pharmacovigilance into routine maternal health services [32, 33]. However, the Malawi PrEP Pregnancy Registry offers several key innovations: integration of PrEP surveillance in maternal health care, use of ScanForm technology to improve data quality, alignment with WHO-recommended pharmacovigilance indicators, and a unique focus on monitoring emerging long-acting PrEP modalities such as CAB-LA and LEN.

A key feature of the registry is integration with existing PrEP documentation systems. Exposure information is not collected in the Registry itself, creating the potential for lost information if records are not matched with a PrEP Visit Card. However, this design allows a new pregnancy registration to be linked to all previous and future PrEP visit information, thereby providing information on the timing of PrEP initiation relative to pregnancy and any PrEP regimen switches during pregnancy. This feature is a significant strength of the Registry design and addresses current gaps in pharmacovigilance systems [8, 16, 19–23]. It also reduces documentation burden for providers, who are already recording PrEP visit information in the existing system. To facilitate linkage to this critical information, ScanForm technology flags records that have not been linked to prompt follow-up with those sites. The Registry is designed as programmatic surveillance with minimal data collection points, meaning it does not collect information on confounders such as PrEP adherence and ANC during pregnancy care or outcomes such as infant health after birth. This limitation is mitigated by the PRI^3^SM-PrIMO Study prospective cohort, which closely follows women through pregnancy and for a year postpartum. Such a hybrid design leverages the scalability of routine data while layering on more detailed research where needed. Finally, because the registry is designed to rely on routine facility-based data, some variability in data quality and completeness across sites is anticipated. To mitigate this, the registry evaluation includes assessment of reach and fidelity, systematic monitoring of data missingness, and provision of ongoing feedback to facilities to support continuous data quality improvement. The use of ScanForm further strengthens data integrity by minimizing missingness and reducing transcription errors through near-real-time error flagging during data capture.

The PrEP Pregnancy Registry represents a novel model of embedding pharmacovigilance for PrEP directly into routine public health systems, resulting in a comprehensive, sustainable and scalable platform for safety surveillance. Following the pilot phase, implementation evaluation will inform the scale-up of the Registry across Malawi, with significant relevance for the development of similar systems across Southern Africa. Evidence from the PrEP Pregnancy Registry will facilitate the safe expansion of CAB-LA and other emerging PrEP modalities, broadening PrEP options for pregnant and breastfeeding women and providing a critical step towards eliminating vertical HIV transmission in Malawi.

## Data Availability

There is currently no dataset used for the current manuscript. However, the registry template as attached in this manuscript and registry enrolment updates are available from the corresponding author on reasonable request.

## Abbreviations

ANC: Antenatal Care
APR: Antiretroviral Pregnancy Registry
CAB: LA–Long Acting Cabotegravir
DVR: Dapivirine Vaginal Ring
EDD: Estimated Delivery Date
FAD: Food and Drug Administration
HIV/AIDS: Human Immunodeficiency Virus / Acquired Immunodeficiency Syndrome
HTS: HIV Testing Services
LEN: Lenacapavir
LMICs: Low–and Middle–Income Countries
MoH: Ministry of Health and Sanitation
NICHD: National Institute of Child Health and Human Development
OCR: Optical Character Recognition
PII: Personally Identifiable Information
PrEP: Pre–Exposure Prophylaxis
PrIMO: Pregnancy, Infant and Maternal Health Outcomes
PRI^3^SM: Preventing Infant Infection with Implementation Science in Malawi
QED: Quantitative Engineering Design
SOP: Standard Operating Procedures
TDF/FTC or 3TC: Tenofovir Disoproxil Fumarate/Emtricitabine
ToT: Training of Trainers
WHO: World Health Organization
